# Asymmetry in Drug Permeability through the Cornea

**DOI:** 10.3390/pharmaceutics13050694

**Published:** 2021-05-11

**Authors:** Nadia Toffoletto, Anuj Chauhan, Carmen Alvarez-Lorenzo, Benilde Saramago, Ana Paula Serro

**Affiliations:** 1 Centro de Química Estrutural, Instituto Superior Técnico, University of Lisbon, Av. Rovisco Pais, 1049-001 Lisbon, Portugal; b.saramago@tecnico.ulisboa.pt (B.S.); anapaula.serro@tecnico.ulisboa.pt (A.P.S.); 2 Centro de Investigação Interdisciplinar Egas Moniz, Instituto Universitário Egas Moniz, Quinta da Granja, Monte de Caparica, 2829-511 Caparica, Portugal; 3 Department of Chemical Engineering, Colorado School of Mines, Golden, CO 80401, USA; chauhan@mines.edu; 4 Departamento de Farmacología, Farmacia y Tecnología Farmacéutica, I+D Farma (GI-1645), Facultad de Farmacia and Health Research Institute of Santiago de Compostela (IDIS), Universidade de Santiago de Compostela, 15782 Santiago de Compostela, Spain; carmen.alvarez.lorenzo@usc.es

**Keywords:** cornea, drug permeability, drug accumulation, bromfenac sodium, dexamethasone sodium

## Abstract

The permeability through the cornea determines the ability of a drug or any topically applied compound to cross the tissue and reach the intraocular area. Most of the permeability values found in the literature are obtained considering topical drug formulations, and therefore, refer to the drug permeability inward the eye. However, due to the asymmetry of the corneal tissue, outward drug permeability constitutes a more meaningful parameter when dealing with intraocular drug-delivery systems (i.e., drug-loaded intraocular lenses, intraocular implants or injections). Herein, the permeability coefficients of two commonly administered anti-inflammatory drugs (i.e., bromfenac sodium and dexamethasone sodium) were determined *ex vivo* using Franz diffusion cells and porcine corneas in both inward and outward configurations. A significantly higher drug accumulation in the cornea was detected in the outward direction, which is consistent with the different characteristics of the corneal layers. Coherently, a higher permeability coefficient was obtained for bromfenac sodium in the outward direction, but no differences were detected for dexamethasone sodium in the two directions. Drug accumulation in the cornea can prolong the therapeutic effect of intraocular drug-release systems.

## 1. Introduction

The eye structure is endowed with efficient protective barriers and mechanisms, which constitute a challenging obstacle to ophthalmic drug delivery [1]. The efficiency of drug administration through eye drops is limited by several factors, including low corneal permeability, drug loss with lacrimation and scarce patient compliance to the treatment, especially when elderly patients are involved [2,3]. As a low drug residence time on the cornea is experienced with eye drops, frequent drop administration is required. In the last decade, progress has been made in the development of alternative drug delivery vehicles, namely drug-loaded contact lenses (CLs) and intraocular lenses (IOLs), able to sustain drug release over time in the cornea and in the anterior chamber, respectively [4,5,6]. Drug-loaded IOLs, in particular, could substitute the prophylaxis after cataract surgery in a patient-friendly and more efficient way, as they overcome the corneal barrier by delivering drugs directly inside the eye.

After the implantation of a drug-loaded IOL, the aqueous humor renovation rate and the drug permeability through the cornea have a major impact on the drug availability over time in the target tissues. The eventual accumulation of the drug released from the IOL in the cornea may constitute an advantage to treat both the anterior and posterior segments of the eye. The permeability values through the cornea, specific for each drug, found in previous literature [7,8,9] are generally obtained experimentally by performing permeability tests with isolated corneas in Franz cells or Ussing chambers.

**Figure 1:**
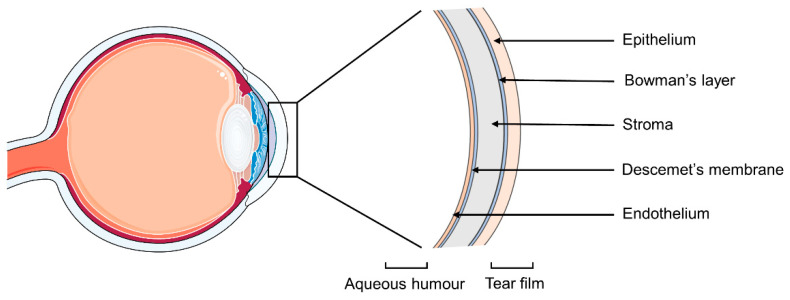
Schematic representation of the layers constituting the cornea.

If literature values are used, the methodology adopted for the test should be carefully considered since the cornea has an asymmetrical structure (Figure 1). The epithelium is the outer layer of the tissue and acts as a protection from the external environment, forming a barrier for chemicals, microbes and water. It has a single layer of basal cells capable of mitosis, and 4–6 layers of wing and surface epithelial cells with tight intracellular junctions. The Bowman’s membrane is composed of collagen and proteoglycans, and helps maintaining the corneal shape. The stroma is the thickest layer of the tissue but also the most hydrophilic one, and it is responsible for the structural support and refractive power of the cornea. Its main components are collagen, keratocytes and glycosaminoglycans. The Descemet’s membrane is the basement layer for the endothelium [10], which is a cell monolayer with a high density of ion transport systems. It regulates the water content of the cornea, preserving its optical transparency [11]. Usually, the corneal permeability of topical drugs is assessed by placing a drug solution in the donor chamber of the Franz cell in direct contact with the underlaying corneal epithelium, in order to simulate the drug flux from the exterior of the eye to the aqueous humor. This set-up is also suitable in case of drug release from contact lenses. However, in case of drug-loaded IOLs, the drug may permeate the cornea in the opposite direction, and a more appropriate Franz cell set-up would involve the corneal endothelium facing the donor chamber. The same reasoning can be applied to any drug administration performed by intraocular injection or drug-eluting intraocular implants.

Despite recent advances in the field, drug release profiles obtained through *in vitro* tests can scarcely reproduce the *in vivo* conditions to which drug-loaded IOLs or implants are subjected in the eye. Previous studies introduced medium renovation in the *in vitro* release tests from IOLs to simulate the aqueous humor renovation [12]. More complex systems, such as microfluidic chambers [13], have also been suggested to simulate the renovation rate. The use of mathematical and numerical simulations has gained interest as a fast and accurate solution for the estimation of the drug release from ocular implants or intraocular injections *in vivo* [14,15]. Pimenta *et al.* [16] developed a mathematical model to predict the drug concentration in the aqueous humor after the implantation of a drug-loaded IOL. Input data of this model consist of the drug release profile obtained *in vitro* under sink conditions, anatomical and physiological parameters of the eye (i.e., aqueous humor volume, renovation rate), the partition coefficient of the drug between the IOL material and the loading solution, the IOL volume and the drug permeability through the cornea. The drug permeability values obtained in the inward direction may not adequately represent the actual *in vivo* conditions, and their use would, therefore, add an error in the model estimation of the drug release profile *in vivo*. To the best of our knowledge, there are no data on outward permeability of ocular drugs.

**Figure 2:**
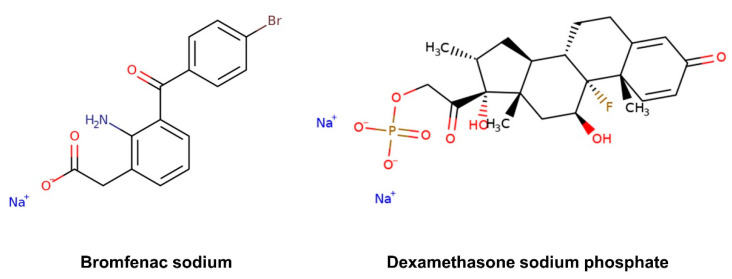
Chemical structure of the selected ophthalmic drugs: bromfenac sodium (MW 356.15 g/mol) and dexamethasone sodium phosphate (MW 516.42 g/mol).

Nonsteroidal anti-inflammatory drugs (NSAIDs) and corticosteroids are currently used to prevent or treat inflammation associated to various ocular conditions, such as scleritis and episcleritis, uveitis and seasonal allergic conjunctivitis, and to alleviate post-operative pain, inflammation and photophobia after corneal refractive surgery [17,18,19]. They also inhibit the inflammatory process of diseases affecting the retina, such as age-related macular degeneration, diabetic macular edema and retinal vein occlusion [20]. Bromfenac sodium, an NSAID, and dexamethasone sodium, a corticosteroid drug, are generally used singularly or in combination for the prevention of chronic inflammation and cystoid macular edema after cataract surgery [17,21]. The objective of this work is to determine the permeability coefficients of these two ophthalmic drugs, which present quite different structural features in terms of chemical functionalities and molecular size (Figure 2), in both inward and outward directions of the cornea, and identify any possible asymmetry in the corneal property. The corneal permeability was evaluated separately for each drug and in the presence of both drugs.

## 2. Materials and Methods

### 2.1. Materials

Dexamethasone sodium phosphate (CAS 2392-39-4) and bromfenac sodium (CAS 91714-93-1) were purchased from Carbosynth (Compton, UK). Phosphate buffer saline (PBS pH 7.4) was prepared with the following composition: KCl 2.7 mM (Scharlau, Barcelona, Spain), NaCl 137 mM (Labkem, Barcelona, Spain), KH_2_PO_4_ 1.8 mM (ITW Reagents—Barcelona, Spain) and Na_2_HPO_4_ 10 mM (Scharlau, Barcelona, Spain). Phosphate buffer (pH 6) was prepared with the following composition: NaOH 1.15 mM (VWR, Paris, France) and KH_2_PO_4_ 10 mM. Porcine eyes were provided by a local slaughterhouse (Compostelana de Carnes S.L.—Santiago de Compostela, Spain), transported immersed in PBS solution in an ice bath and used within three hours from eye collection.

### 2.2. Permeability Test

**Figure 3:**
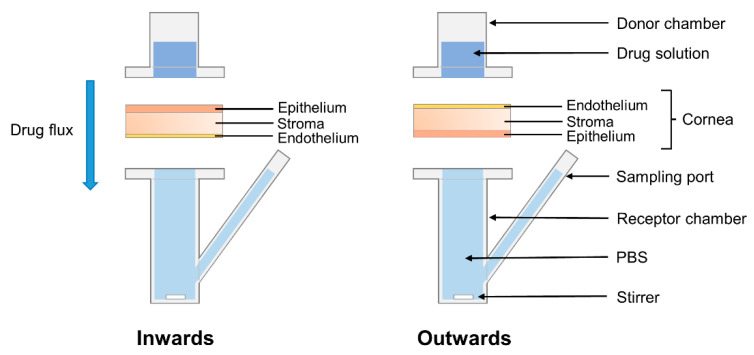
Schematic representation of the Franz cells configurations used for the permeability test. The cornea was positioned with the epithelium facing upwards to evaluate drug permeability inwards, or with the endothelium facing upwards to evaluate drug permeability outwards.

Corneas were isolated from porcine eyes maintaining 2–3 mm of surrounding sclera for support, then rinsed in PBS and mounted on vertical diffusion Franz cells in two different configurations (Figure 3) as follows. Half of the corneas (*n* = 9) were placed with the epithelium facing upwards to evaluate drug permeability inwards, while the other half (*n* = 9) were placed with the endothelium facing upwards to evaluate drug permeability outwards. After clamping, a corneal surface area of 0.785 cm^2^ was in direct contact with both the donor and receptor chambers, which corresponds to the internal sectional area of the Franz cells. Donor and receptor chambers were filled with PBS (1 mL and 6 mL, respectively). Franz cells were then placed in a bath at 37 °C with magnetic stirring of the receptor chamber to balance the tissues.

After 30 min, the volume of the donor chamber was substituted by 1 mL of drug solution. Three different drug solutions were prepared in PBS: a dual-drug solution with bromfenac sodium 125 µg/mL and dexamethasone sodium 125 µg/mL, a single-drug bromfenac sodium 125 µg/mL solution and a single-drug dexamethasone sodium 125 µg/mL solution. The receptor chamber was covered with parafilm to prevent evaporation. At each time point (i.e., 0.5, 1, 2, 3, 4, 5 and 6 h), 1 mL of medium was removed from the receptor chamber for determination of the drug concentration (Section 2.3) and replaced with fresh PBS. All experiments were performed in triplicate.

After 6 h, the donor chamber volume was collected for analysis. Corneas were rinsed and immersed in 2 mL acetonitrile overnight at 36 °C, sonicated for 99 min and centrifuged (5 min at 25 °C and 1000 rpm followed by 20 min at 25 °C and 14,000 rpm). Supernatants were collected and filtered (0.22 µm) after each centrifugation and analyzed as described below (Section 2.3) to quantify the amount of drug.

The cumulative amounts along the time (t) of permeated dexamethasone sodium and bromfenac sodium were calculated and fitted to a linear least squares regression. The steady state flux, J, was obtained from the slope of the regression (Equation (1)), while the lag time, t_lag_, corresponded to the x-intercept. The permeability coefficient, P_coeff_, for each drug and experimental set-up were obtained as the ratio between J and the drug concentration in the donor chamber at t = 6 h (Equation (2)) [22,23,24,25]:

Cumulative mass permeated/Surface area = J t + q(1)

P_coeff_ = J/[Donor]_t=6 h_ (2)

### 2.3. Drug Quantification

The amount of drug in the donor and receptor solutions was quantified using a Waters HPLC apparatus fitted with a 717 Plus Autosampler system and a UV detector. A SunFire C18 4.6 × 150 mm column with 4.5 µm pores (Waters, Ireland) was selected. The analysis was performed at room temperature (25 °C) with an 80 µL injection volume and a flow rate of 1 mL/min. The mobile phase consisted in a mixture of 0.01 M phosphate buffer (pH 6.0) [26] and acetonitrile in the ratio of 72:28 *v/v*. Dexamethasone sodium and bromfenac sodium were quantified at 242 nm and 265 nm, respectively. The retention time of dexamethasone sodium and bromfenac sodium was 4.20 min and 14.65 min, respectively. Two calibration curves were prepared to quantify the drugs in different concentration ranges in PBS (i.e., 0.05–10 µg/mL and 25–500 µg/mL). The curves were prepared in triplicate and validated with respect to linearity, accuracy and precision. All samples were filtered (0.22 µm) before testing. The quantification limits of the method were 7 ng/mL and 27 ng/mL for dexamethasone sodium and bromfenac sodium, respectively.

### 2.4. Statistical Analysis

Data are presented as mean ± standard deviation. Statistical analysis was performed by *t*-test to compare two datasets (namely, data obtained in inward vs. outward configuration and data obtained with a single-drug vs. dual-drug solution in the donor chamber). GraphPad Prism version 8.0.0 for Windows (GraphPad Software, San Diego, CA, USA) was used. Significance level was set at *p* < 0.05.

## 3. Results

**Figure 4:**
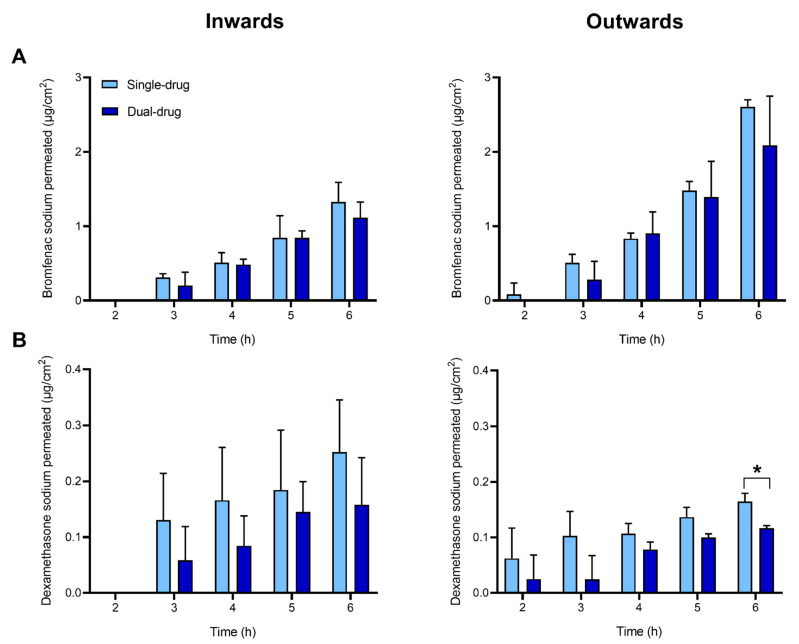
Cumulative amount of drug permeated through the cornea over time: bromfenac sodium (**A**) and dexamethasone sodium (**B**). Single- or dual-drug solutions with 125 µg/mL of each drug were placed in the donor chamber. Experiments were conducted in inward (left column) or outward (right column) flux direction through the cornea. Mean values and standard deviations (*n* = 3); * *t*-test; *p* < 0.05.

Permeability inward and outward the cornea of bromfenac sodium and dexamethasone sodium was investigated *ex vivo* using vertical Franz diffusion cells over a 6-h time span. The experiments were performed with single- and dual-drug solutions to investigate whether the concomitant presence of both drugs may have any effect on the permeability of each drug. Cumulative drug amounts permeated through the cornea are reported in Figure 4, Table 1 and Supplementary Table S1.

After 6 h, the amount of permeated bromfenac sodium from single-drug solution was significantly higher in the outward direction (2.61 ± 0.09 µg/cm^2^) than in the inward direction (1.3 ± 0.3 µg/cm^2^). This tendency was evidenced in the values of the steady-state flux J (Table 1), obtained by linear regression of the cumulative mass permeated over time (Supplementary Figure S1), and in the calculated permeability coefficients (8 × 10^−7^ and 19 × 10^−7^ cm/s in inward and outward direction, respectively) (Table 1). The lag time was about 2 h in both directions. No statistical difference was detected in the permeability parameters (i.e., cumulative mass permeated, J and P_coeff_) obtained for bromfenac sodium in a single-drug solution when compared to the dual-drug solution.

The cumulative amounts of dexamethasone sodium permeated through the cornea both in the inward and outward directions (0.12–0.25 µg/cm^2^) were one order of magnitude lower than those recorded for bromfenac sodium (Figure 4, Table 1). This tendency was evidenced in the values of the flux J and in the permeability coefficient (0.7–0.9 × 10^−7^ cm/s). As opposed to the results obtained for bromfenac sodium, no significant differences were observed between the permeability values of dexamethasone sodium inwards and outwards the cornea. A statistical difference between the single-drug dexamethasone solution and the dual-drug solution was detected only in the drug amount permeated in outward direction at t = 6 h, but this difference was not reflected in the calculated permeability coefficients. All calculations were done assuming a pseudo-steady state. The validity of this assumption will be discussed later.

**Figure 5:**
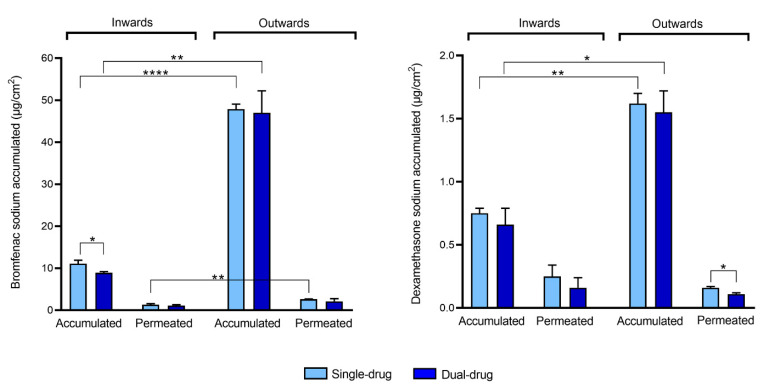
Bromfenac sodium (**left**) and dexamethasone sodium (**right**) accumulated in the cornea and permeated to the receptor chamber (normalized per unit area) in an inward or outward corneal configuration when tested as a single-drug solution (Single-drug) or dual-drug solution (Dual-drug). Mean values and standard deviations (*n* = 3); *t*-test * *p* < 0.05; ** *p* < 0.01; **** *p* < 0.0001.

The amount of drug accumulated in the cornea was quantified at the end of the permeability test (Figure 5, Supplementary Table S1). In the outward direction, the amount of bromfenac sodium accumulated in the cornea (47–48 µg/cm^2^) was almost five times higher compared to the drug accumulated when the tissue was tested in the inward flux direction (9–11 µg/cm^2^). Similarly, a significantly higher amount of dexamethasone was detected in the cornea when tested in the outward direction (1.5–1.6 µg/cm^2^) if compared to the inward direction (0.6–0.7 µg/cm^2^). These results are in agreement with the higher permeability values of bromfenac sodium in the outward direction, but they are apparently in contradiction with the fact that no difference was encountered between the permeability values of dexamethasone in both directions (Table 1). A statistical difference in the drug amount accumulated in the cornea when tested as single-drug solution or dual-drug solution was observed only for bromfenac sodium in the inward direction; bromfenac accumulation was higher when the single-drug solution was tested.

## 4. Discussion

Several diffusion cells (e.g., vertical Franz cells, Ussing chambers, horizontal perfusion cells with a convex surface shape [27]) have been proposed to perform *ex vivo* test on corneal tissues, and modifications were designed to better mimic the physiological curvature of the cornea after excision [28,29] or to preserve the integrity of the tissue by CO_2_ and O_2_ bubbling [30]. However, classic Franz cells still remain widely used in the field [22,31,32] as a low-cost and simple equipment. During the excision of the corneas from porcine eyes, 2–3 mm of surrounding sclera was maintained to be used as support. To limit the distortion of the corneal shape in the Franz cells, clamping mainly addressed the external scleral portion of the tissue and was carefully performed, avoiding excessive stretch in the corneas. Eventual bubbles formed during sampling from the receptor chamber at each time point were removed. The possibility to preserve the corneal integrity even in absence of oxygenation for the standard duration of *ex vivo* permeability tests has been demonstrated in previous literature [27,33]. In particular, Pescina *et al.* [27] investigated the use of Franz cells in absence of O_2_ and CO_2_ bubbling for 5 h. Although a detachment of the most apical layers of the epithelium was observed after 5 h of *ex vivo* test, the basal side of epithelium remained intact without signs of edema in the tissue. The reported permeability data were compared with literature values obtained using more complex set-ups with bubbling, and the goodness and robustness of the model were confirmed.

The possibility of self-association forming different structures in aqueous solution may also influence the permeability of amphiphilic drugs [34,35]. Due to the presence of fused aromatic rings, dexamethasone sodium phosphate does not form micelles but undergoes open or continuous association above its critical association concentration (CAC = 6.91 mM) [36]. Diclofenac, an NSAID with a similar molecular structure to bromfenac sodium, has been reported to form micellar structures above the concentration of 35 mM [37]. The maximum drug concentration herein used was 125 µg/mL for both bromfenac sodium and dexamethasone sodium, which corresponds to 0.35 mM and 0.24 mM, respectively. As this concentration is far below the CAC of dexamethasone sodium and diclofenac found in literature, it is assumed that both drugs were present as individual molecules in the aqueous medium.

No significant difference was found in the permeability coefficients obtained with a single-drug solution if compared with a dual-drug solution of bromfenac and dexamethasone, suggesting a lack of association between the two drugs during the permeation through the tissue, as expected from their similar charges and assuming passive diffusion mechanism.

A comparison of the amounts of drug accumulated in the cornea and permeated to the receptor chamber (Figure 5, Supplementary Table S1) revealed that, in both directions, the amounts of bromfenac sodium and dexamethasone sodium accumulated in the cornea were higher than those which permeated. Moreover, a significantly higher drug accumulation in the cornea was obtained in the outward direction for the two drugs, which is consistent with the multilayered structure of the cornea. The inner and outer layers, namely the endothelium and epithelium, have different functions. The epithelium blocks the passage of foreign material inside the eye, while the endothelium controls the net flux of ions from the cornea to the aqueous humor, thus avoiding corneal edema [11]. As the cornea is non-vascularized, another fundamental function of the endothelium is to allow the transport of nutrients from the aqueous humor to the external corneal layers [38]. Therefore, in inward configuration, drug accumulation from the donor chamber should be hindered by the presence of the slightly permeable epithelium.

Drug accumulation mechanisms in the corneal layers were previously addressed by a few groups [39,40], but remain in need of further investigation. Interestingly, the study of Hsu *et al.* [40] on timolol and dorzolamide-loaded CLs for the management of glaucoma in Beagle dogs evidenced a lowering of intraocular pressure for a further 8 days after the removal of the therapeutic lenses. The authors hypothesized an intracellular drug accumulation during the prolonged CL wear (4 days), followed by drug diffusion from the cornea after lens removal. Although the dependence of the kinetics of drug release from the corneal layers on the layer of major accumulation (e.g., epithelium in case of drug exposure inwards and endothelium/stroma in outward direction) and the transport mechanism (e.g., paracellular or transcellular) must be further investigated, the ability of the cornea to act as a drug reservoir should be considered due to its relevant clinical advantages. If compared to eye drops or injections, drug-eluting implants [41,42,43] and therapeutic ophthalmic lenses [16,44] provide sustained ocular delivery and prolong the exposure time of the tissues to the drugs. As a result, drug accumulation in the cornea is favored and its subsequent release could potentially result in a more prolonged therapeutic efficacy of the devices even after their exhaustion.

In outward corneal configuration, the drug concentration in the stroma will tend to equilibrate to the drug concentration in the donor chamber, due to the negligible barrier effect of the endothelium. The partition coefficient K of the drug between the stroma and the donor chamber can be obtained as the ratio between the drug concentration in the stroma and in the donor chamber at equilibrium (Equation (3)):

K = [stroma]_t=6 h_/[donor chamber]_t=6 h_ (3)

As the stroma constitutes more than 90% of the corneal thickness [45], it is reasonable to approximate the drug amount accumulated in the stroma with the drug amount accumulated in the cornea. Considering the amount of bromfenac sodium accumulated in the cornea at t = 6 h (Figure 5), the thickness of the porcine stroma (≈ 600 µm [46]) and the area exposed to the drug in the Franz cell (0.785 cm^2^), the drug concentration in the stroma resulted equal to 785 µg/mL. The concentration of bromfenac sodium detected in the donor chamber at t = 6 h resulted equal to ≈ 78 µg/mL in outward configuration (Supplementary Table S1), leading to K ≈ 10.

The lag time t_lag_ needed to reach a steady state flux across the cornea is related to tissue thickness, H, by Equation (4) [47] and is, therefore, mainly controlled by the stroma [48] with H = 600 µm [46]. Then, the lag time t_lag_ ≈ 2.3 h (Table 1) leads to the diffusivity of bromfenac sodium through the stroma D = 7 × 10^−12^ m^2^/s. The permeability coefficient of the stroma can be obtained from Equation (5) [47]:

t_lag_ = H^2^/ (6 D) (4)

P_coeff_ = D K/H (5)

The resulting P_coeff_ of bromfenac sodium in the stroma is 116 × 10^−7^ cm/s, which is one order of magnitude higher than the values obtained for the entire corneal tissue (Table 1). Therefore, the resistance of the stroma to permeability was negligible, thus confirming that the epithelium constitutes the limiting element in the permeation of hydrophilic drugs. As a consequence, the driving force for permeability is the difference in concentration across the epithelium. In outward configuration, this difference occurs between the drug concentration in the stroma and in the receptor chamber. As K ≈ 10, the drug concentration in the stroma is about 10 times higher than in the donor chamber. In inward configuration, the epithelium faces the donor chamber above and the stroma below, which, due to its high diffusivity and high permeability coefficient, tends to equilibrate to the low drug concentration (≈0) of the receptor chamber. As a result, the driving force for permeability across the epithelium in outward configuration is about 10 times higher than in inward configuration, which justifies the asymmetric behavior of the tissue: in fact, significantly higher values of the cumulative amount of drug permeated, J and P_coeff_ of bromfenac sodium were obtained when the cornea was tested in outward configuration (Table 1). According to the calculated K, a 10-fold higher flux across the cornea was expected in outward configuration if compared to inward configuration, but only a doubled J value was experimentally obtained, which leads to the hypothesis that bromfenac sodium could bind to the stroma with a consequent reduction in the drug amount available for permeation.

The high permeability coefficient of the stroma implies that it has a low drug concentration when placed in contact with the receptor chamber; thus, in inward configuration, drug accumulation mainly occurs in the epithelium. The lower amount of drug accumulated in inward direction is consistent with the slow accumulation occurring in the epithelium, also reported in previous literature [39].

Contrarily to what was found with bromfenac sodium, this asymmetric corneal behavior was not observed in the permeability parameters obtained for dexamethasone sodium. However, it should be noted that the amount of dexamethasone quantified in the receptor chamber of the diffusion cells at each time point was significantly lower, with the analyzed concentrations being close to the minimum quantification limit of the chromatographic method (i.e., 7 ng/mL). As a consequence, a high standard deviation and lower R^2^ values were associated to the obtained results and to the linear regression of the cumulative mass versus time, respectively (Table 1, Supplementary Figure S2), which could have masked the differences in the permeability values in the two different flux directions. Moreover, the lag time associated to dexamethasone sodium was t_lag_ ≈ 0 (Table 1), which may indicate that data for dexamethasone sodium were collected in the initial unsteady portion of the permeated drug over time curve (Supplementary Figure S2), and that it was not possible to achieve a steady state after 6 h of *ex vivo* test. This can be confirmed with the calculation of the partition coefficient K of dexamethasone in the stroma in the outward configuration (Equation (3)). Drug concentration in the donor chamber at t = 6 h resulted equal to ≈95 µg/mL in the outward direction (Supplementary Table S1). Considering the volume of the analyzed tissue (≈0.047 cm^3^) and the amount of dexamethasone sodium accumulated in the cornea (Figure 5), drug concentration in the stroma at t = 6 h can be approximated to 26 µg/mL, which results in K ≈ 0.28. As the stroma is a water-rich tissue, the K value for hydrophilic drugs is expected to be ≥1. Therefore, the obtained K for dexamethasone suggests that the stroma did not reach an equilibrium with the donor chamber by the end of the test, and that all permeability data related to dexamethasone sodium were collected in unsteady state.

Permeability values reported in the literature for *ex vivo* corneal permeability vary in a wide range between 0.7 and 110 × 10^−7^ cm/s depending on the drug and the experimental conditions [9,24]. The values here obtained for both dexamethasone sodium and bromfenac sodium lie in this same interval but in the smallest values region, which can be justified by the hydrophilic nature of these two drugs [49]. In fact, the drug transfer through the epithelium, which acts as a lipid-like barrier, was demonstrated to be the limiting step for hydrophilic drugs in a rabbit model [50]. As the calculated P_coeff_ of dexamethasone sodium was obtained in an unsteady state, the obtained values are not quantitatively reliable. However, it is possible to state that the permeability of the cornea to dexamethasone is lower than to bromfenac sodium, especially in the outward direction. Dexamethasone sodium has a stronger acid moiety (pK_a_ 1.89 and 6.4 for the two potentially charged groups [51]) than bromfenac sodium (pK_a_ 4.29 [52]). When dissolved in PBS at pH 7.4, about 0.0002% of dexamethasone and 0.0740% of bromfenac sodium are expected to be in their nonionized form, according to the Henderson–Hasselbalch equation (Equation (6)). As ionized drugs are minimally lipid soluble, passive diffusion of dexamethasone through the cornea is not favored at all [53], thus justifying the low permeability values encountered and the difference in behavior between the two drugs. The larger molecular weight of dexamethasone sodium (516.42 g/mol) compared to bromfenac sodium (356.15 g/mol) and the presence of two charged groups at pH 7.4 instead of the single-charge of bromfenac (Figure 2) may also have contributed to the difference in permeability, as moderately-charged small molecules are expected to permeate the cornea more easily [9,54].

pK_a_ − pH = log ([nonionized]/[ionized]) (6)

The obtained results may indicate that the barrier effect of the endothelium becomes significant in the case of the highly hydrophilic and highly acidic drugs, such as dexamethasone sodium. In fact, even if its resistance to permeability is usually neglected due to its low thickness [39], the endothelium is lipophilic [55]. As a result, even if the resistance of the epithelium remains the main barrier to drug permeability, dexamethasone accumulation into the cornea (≈1.5 µg/cm^2^) is much lower than for bromfenac (≈47 µg/cm^2^) even in the outward configuration.

## 5. Conclusions

The corneal permeation of bromfenac sodium and dexamethasone sodium, respectively an NSAID and a corticosteroid, commonly administered as ophthalmic anti-inflammatory drugs, was assessed in both inward and outward directions. Permeability tests evidenced a higher drug accumulation in the cornea when the drug solution is applied in an outward corneal configuration. This can be justified by the higher permeability of the endothelial layer if compared to the epithelial layer of the tissue. Coherently, the permeability value of bromfenac sodium was higher in the outward direction (19–21 × 10^−7^ cm/s) than in the inward direction (7–8 × 10^−7^ cm/s). However, no difference was detected for the permeability coefficient of dexamethasone sodium in the two directions (0.7–0.9 × 10^−7^ cm/s), possibly due to the HPLC detection limit. No difference in permeability values was found comparing the presence of a single-drug or dual-drug solution in the donor chamber. As a conclusion, in case of drug-eluting intraocular lenses, intracameral injections or implants, the asymmetry of the corneal permeability must be considered for an appropriate estimation of the drug loss through the tissue. Moreover, the high accumulation of drugs in the cornea may contribute to prolong the drug supply to other tissues.

## Figures and Tables

**Table 1:** Cumulative mass permeated after 6 h, lag-time (t_lag_), steady state flux (J) and permeability coefficient (P_coeff_) of the drugs when tested as a single-drug solution (Single) or dual-drug solution (Dual).

	Flux Direction	Drug Solution	Cumulative Mass Permeated after 6 h (µg/cm^2^)	t_lag_ (h)	J (µg/(cm^2^ h))	P_coeff_ × 10^7^ (cm/s)	R^2^
Bromfenac sodium	Inwards	Single	1.3 ± 0.3	2.3 ± 0.2	0.34 ± 0.09	8 ± 2	0.94 ± 0.04
		Dual	1.1 ± 0.2	2.3 ± 0.5	0.31 ± 0.05	7 ± 1	0.97 ± 0.02
	Outwards	Single	2.61 ± 0.09	2.1 ± 0.2	0.55 ± 0.08	19 ± 3	0.94 ± 0.02
		Dual	2.1 ± 0.7	2.4 ± 0.4	0.59 ± 0.26	21 ± 9	0.97 ± 0.01
Dexamethasone Sodium *	Inwards	Single	0.25 ± 0.09	0	0.038 ± 0.006	0.9 ± 0.1	0.9 ± 0.1
		Dual	0.16 ± 0.08	1 ± 1	0.04 ± 0.01	0.8 ± 0.3	0.8 ± 0.2
	Outwards	Single	0.16 ± 0.01	0	0.02 ± 0.01	0.7 ± 0.3	0.8 ± 0.2
		Dual	0.12 ± 0.01	0.1 ± 4	0.03 ± 0.01	0.8 ± 0.4	0.87 ± 0.07

* All calculations are done assuming a pseudo-steady state. The validity of this assumption is discussed below.

## Data Availability

The authors confirm that the data supporting the findings of this study are available within the article and its supplementary materials.

## Supplementary Materials

The following information is available online at https://www.mdpi.com/article/10.3390/pharmaceutics13050694/s1: Figure S1: Linear regression of the cumulative mass of bromfenac sodium permeated through the cornea in inward (left) or outward (right) direction. The experiment was conducted in the presence of bromfenac sodium and dexamethasone simultaneously (dual drug) or with bromfenac sodium alone (single drug); Figure S2: Linear regression of the cumulative mass of dexamethasone sodium permeated through the cornea in inward (left) or outward (right) direction. The experiment was conducted in the presence of bromfenac sodium and dexamethasone simultaneously (dual drug) or with dexamethasone sodium alone (single drug); Table S1: Amount of bromfenac sodium and dexamethasone sodium detected in the donor chamber after the test (t = 6 h), accumulated in the cornea and permeated to the receptor chamber. The reported data were summed and compared to the initial drug amount placed in the donor chamber (i.e., 125 µg) for mass balance assessment.
