# Supplementary material for: Asymmetry in Drug Permeability through the Cornea
Source: Pharmaceutics. 2021 May 11;13(5):694. doi: 10.3390/pharmaceutics13050694 (PMC8151369; doi:10.3390/pharmaceutics13050694)
Supplement: Supplementary file 1 [file pharmaceutics-13-00694-s001.zip › pharmaceutics-1145160-supplementary.pdf]

pharmaceutics

MDPI

Supplementary Materials: Asymmetry in Drug Permeability through the Cornea

Nadia Toffoletto, Anuj Chauhan, Carmen Alvarez-Lorenzo, Benilde Saramago and Ana Paula Serro

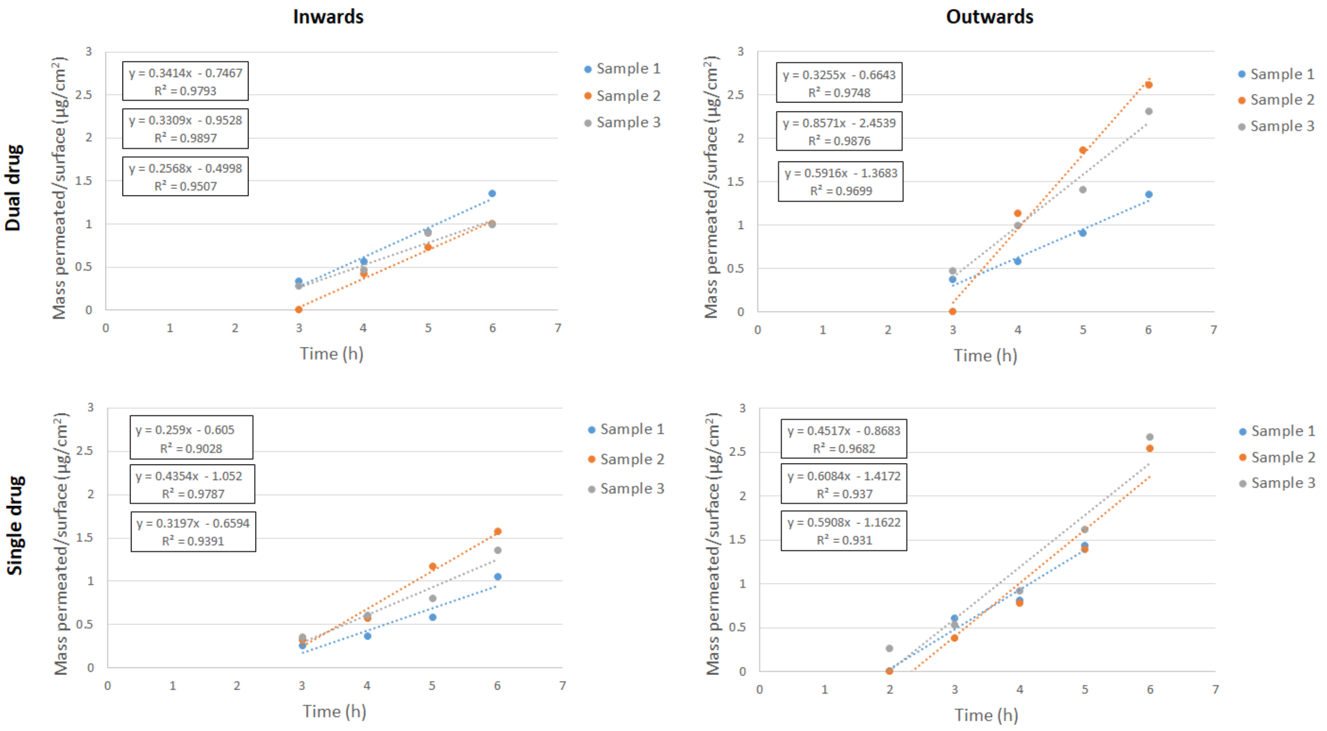

Figure S1. Linear regression of the cumulative mass of bromfenac sodium permeated through the cornea in inward (left) or outward (right) direction. The experiment was conducted in presence of bromfenac sodium and dexamethasone simultaneously (dual drug) or with bromfenac sodium alone (single drug).

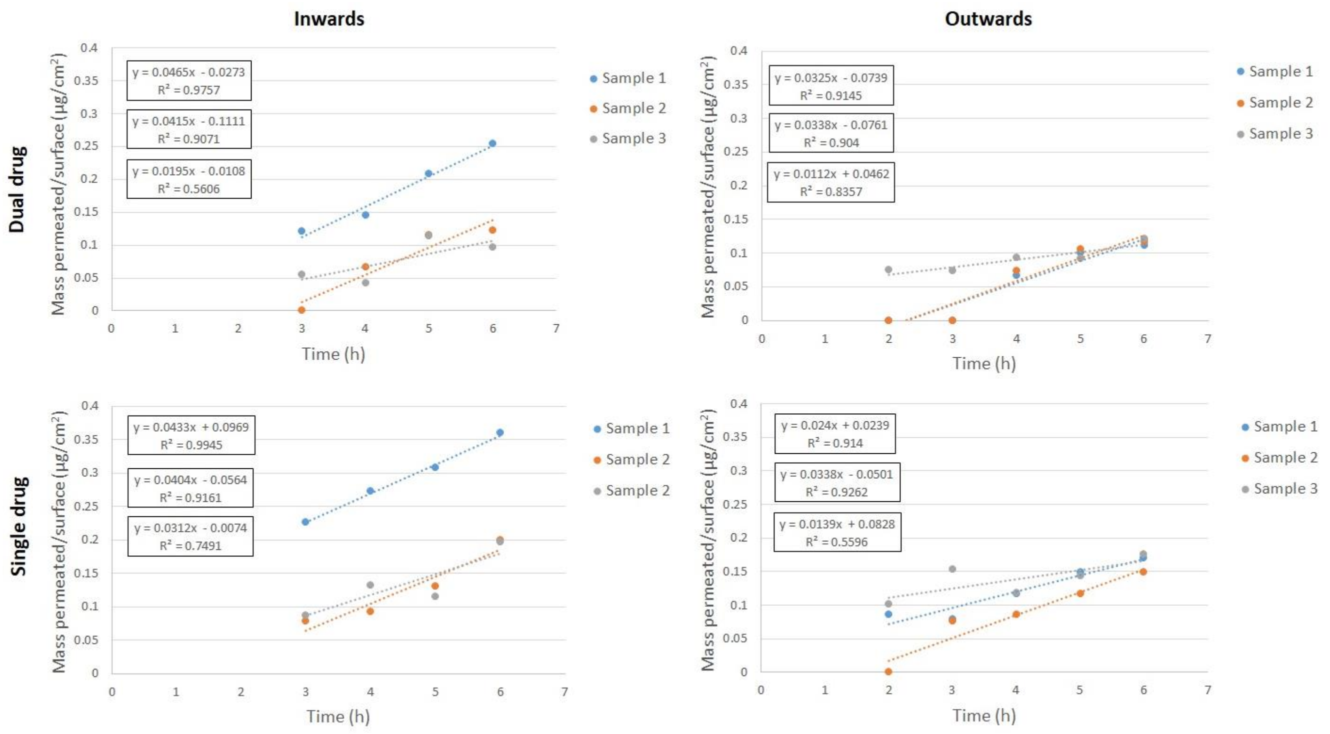

Figure S2. Linear regression of the cumulative mass of dexamethasone sodium permeated through the cornea in inward (left) or outward (right) direction. The experiment was conducted in presence of bromfenac sodium and dexamethasone simultaneously (dual drug) or with dexamethasone sodium alone (single drug).

Table S1. Amount of bromfenac sodium and dexamethasone sodium detected in the donor chamber after the test (t = 6 h), accumulated in the cornea and permeated to the receptor chamber. The reported data were summed (Total drug amount) and compared to the initial drug amount placed in the donor chamber (i.e. 125 µg) for mass balance assessment.

		**Bromfenac sodium**		**Dexamethasone sodium**	
		Inwards	Outwards	Inwards	Outwards
**Single-drug solution**	Drug in the donor chamber (t = 6 h)	112 ± 1 µg	78 ± 6 µg	122 ± 2 µg	98.68 ± 0.07 µg
	Drug accumulated in the cornea	8.7 ± 0.6 µg	37.6 ± 0.9 µg	0.59 ± 0.03 µg	1.27 ± 0.06 µg
	Drug permeated to the receptor chamber	1.0 ± 0.2 µg	2.05 ± 0.07 µg	0.20 ± 0.07 µg	0.13 ± 0.01 µg
	Total drug amount	≈122 µg	≈119 µg	≈123 µg	≈100 µg
**Dual-drug solution**	Drug in the donor chamber (t = 6 h)	114 ± 3 µg	78 ± 2 µg	122 ± 2 µg	93 ± 3 µg
	Drug accumulated in the cornea	7.0 ± 0.2 µg	37 ± 4 µg	0.5 ± 0.1 µg	1.2 ± 0.1µg
	Drug permeated to the receptor chamber	0.9 ± 0.2 µg	1.6 ± 0.5 µg	0.12 ± 0.06 µg	0.091 ± 0.004 µg
	Total drug amount	≈122 µg	≈117 µg	≈122 µg	≈95 µg
